# Artificial Intelligence for the Food Industry

**DOI:** 10.3390/foods15091456

**Published:** 2026-04-22

**Authors:** Malik A. Hussain, Azharul Karim

**Affiliations:** 1 School of Science, Faculty of Engineering, Computing and Science, Western Sydney University, Richmond, NSW 2753, Australia; 2 School of Mechanical, Medical and Process Engineering, Faculty of Science and Engineering, Queensland University of Technology, Brisbane, QLD 4001, Australia; azharul.karim@qut.edu.au

Artificial Intelligence (AI) is transforming the food industry by enhancing food safety (contamination detection, traceability), optimizing supply chains (demand forecasting, waste reduction, logistics), personalizing nutrition (customized recommendations), and driving product innovation (new flavor creation, formulation) through data analysis, machine vision, and predictive analytics, leading to greater efficiency, sustainability, and consumer satisfaction from farm to fork [1,2]. This Special Issue (SI) invited original research, reviews, and opinion articles on current AI technologies in the food industry, focusing on emerging applications, challenges, and innovative solutions. Nine articles were accepted and published in the SI.

Machine learning (ML)-based models have been increasingly adopted by the food processing industry. Hussain et al. [Contribution 1] provided an overview of how ML technologies are transforming the food industry operations such as drying, frying, cooking, heating, and baking for improved productivity, profitability, and sustainability. They also highlighted the applicability of a new ML method, physics-informed machine learning (PIML), in food processing. The contribution by Liakos et al. [Contribution 2] included a systematic review of recent advancements in ML for quality control in the food industry covering key areas such as food quality applications, detection and visual inspection systems, ingredient optimization and nutritional assessment, packaging (sensors and predictive QC), supply chain (traceability and transparency and food industry efficiency) and Industry 4.0 models.

Simonič et al. [Contribution 3] developed a long short-term memory (LSTM)-based predictive model to estimate moisture content in continuous corn drying systems, highlighting the potential of AI for improving energy efficiency and process performance. Another key area addressed in this book is the use of computer vision and automation in food manufacturing. Verk et al. [Contribution 4] presented an application of Mask R-CNN for potato segmentation in sorting systems, achieving precise instance segmentation and demonstrating the feasibility of deploying deep learning models in industrial environments. Similarly, Kim and Kim [Contribution 5] introduced an integrated system combining convolutional neural networks with a six-axis robotic arm for the automation and optimization of coffee roasting. Their system utilizes real-time sensor and image data to control roasting conditions, illustrating how AI-enabled robotics can enhance process precision, consistency, and productivity.

AI applications in consumer engagement and supply chain optimization are also represented in this collection. Tellechea et al. [Contribution 6] have demonstrated that advanced algorithms, such as deep Q-networks, can significantly improve recommendation performance and contribute to reducing food waste by better aligning supply with consumer preferences. Stecuła et al. [Contribution 7] highlighted how digitalization is transforming consumer interactions with food systems, enabling more convenient, personalized, and efficient shopping experiences. These developments illustrate the broader impact of AI across the entire food value chain, from production to consumption. The emergence of data-centric AI approaches is further illustrated by Bölücü et al. [Contribution 8], who investigated the use of large language models (LLMs) to supplement structured datasets and demonstrated that LLMs can effectively extract relevant parameters from the scientific literature, significantly reducing manual data curation efforts.

Quality control remains a critical priority within the food industry, and contributions in this collection address this challenge [3]. Liakos et al. [Contribution 2] provided a comprehensive review of machine learning applications in food quality control, covering domains such as defect detection, predictive quality assessment, and supply chain traceability. Torrico [Contribution 9] explored the novel application of large language models, specifically ChatGPT (version 3.5), as a sensory evaluator for food products. By generating descriptive sensory profiles and sentiment analysis for different chocolate brownie formulations, this study demonstrates the potential of generative AI to support early-stage product development and screening.

Despite these advancements, several challenges remain. Future research should focus on the integration of AI with complementary technologies such as the Internet of Things (IoT), digital twins, blockchain, and advanced sensor systems [1,4]. Such integration will enable the development of fully connected, intelligent, and adaptive food systems capable of real-time optimization and decision-making.

## Data Availability

The data described here are publicly available in the Special Issue “Artificial Intelligence for the Food Industry”.
